# Therapeutic Effects of *Punica granatum* (Pomegranate): An Updated Review of Clinical Trials

**DOI:** 10.1155/2021/5297162

**Published:** 2021-11-16

**Authors:** Samira Eghbali, Sayyedeh Fatemeh Askari, Razieh Avan, Amirhossein Sahebkar

**Affiliations:** ^1^Department of Pharmacognosy, School of Pharmacy, Birjand University of Medical Sciences, Birjand, Iran; ^2^Cardiovascular Diseases Research Center, Birjand University of Medical Sciences, Birjand, Iran; ^3^Department of Phytopharmaceuticals (Traditional Pharmacy), School of Pharmacy, Birjand University of Medical Sciences, Birjand, Iran; ^4^Department of Clinical Pharmacy, Medical Toxicology and Drug Abuse Research Center (MTDRC), School of Pharmacy, Birjand University of Medical Sciences, Birjand, Iran; ^5^Applied Biomedical Research Center, Mashhad University of Medical Sciences, Mashhad, Iran; ^6^Biotechnology Research Center, Pharmaceutical Technology Institute, Mashhad University of Medical Sciences, Mashhad, Iran; ^7^School of Pharmacy, Mashhad University of Medical Sciences, Mashhad, Iran

## Abstract

*Punica granatum* L. belongs to the *Punicaceae* family which is distributed around the world. Different parts of pomegranate like seed, peel, juice, and leaves are rich in potential bioactive compounds. These plants have found application in traditional medicine such as in treatment of gastrointestinal, cardiovascular, and endocrine diseases, among others. The present review aimed to summarize the current research on the traditional and scientific applications of *P. granatum* with regard to the phytochemical content and clinical applications that may be useful for future drug development. Information about *P. granatum* was obtained from local classic herbal literature and electronic databases, such as PubMed, Scopus, and ScienceDirect. Several phytochemical constituents including polyphenolics, flavonoids, anthocyanosides, alkaloids, lignans, and triterpenes have been reported from the plant. Randomized clinical trials have provided evidence as to the pharmacological activities of pomegranate in several diseases including diabetes, cardiovascular disease, oral cavity disorders, endocrine disorders, and cancer. The present review has provided an insight into the traditional applications of the plants, and some of them have been validated by scientific evidence, particularly their applications as treatment of cardiovascular and endocrine diseases.

## 1. Introduction

### 1.1. *Punica granatum* L. (Pomegranate)

*Punica granatum* L. (pomegranate) is a well-known member of *Punicaceae* family, which comprises two species, *Punica granatum* (indigenous to Mediterranean regions and Iran) and *Punica protopunica* (endogenous to Socotra islands). It is widely cultivated throughout Central Asia, the Himalayas, Middle East, American Southwest, and Mediterranean area and is believed to originate from Iran and Afghanistan [1]. The pomegranate tree is a long-living tree that can typically grow up to 12 to 16 feet and live over 200 years. The leaves are glossy and the flowers are red, white, large, or variegated and have tubular calyxes which finally become the fruit. The pomegranate fruit is grenade-shaped with a deep red, leathery skin and crown-shaped calyx. The seeds are surrounded by a small amount of tart and red juice and are separated via white, membranous pericarp [2].

### 1.2. Applications of Pomegranate

Diet is the principal point among various lifestyle modification factors in traditional Persian medicine, i.e., before using the medication; the first choice is food consumption [3]. The nutritional properties of pomegranate have been studied in numerous studies, so it could be a good candidate for food additives and preservatives because of antioxidant and antimicrobial activity, having pectin and fiber. Moreover, waste materials (peels) would be a natural option for food packaging because of plasticizing, strengthening, and elongation activity besides its specific color [4–6]. Pomegranate has been used in traditional medicine for the treatment of diarrhoea, dysentery, hemorrhoids, intestinal parasites, sore throat, diabetes, epistaxis, and vaginal itching and is believed to be tonic for the heart [7, 8]. In addition, it has recently been used in treatment of numerous diseases including diabetes [9], Alzheimer's disease [10], cancer [11, 12], arthritis [13], male infertility [14], obesity [15], and cardiovascular disorders [16]. Many traditional effects of medicinal plants have been proven in modern studies [17, 18], so they can be considered as a source for designing new drug formulations. Currently, the COVID-19 pandemic is considered a global public health concern. There is no particular therapy against COVID-19. Several clinical and preclinical studies are currently performed to investigate a gold-standard therapy with high possible efficacy and low adverse effect [19, 20]. Pomegranate is recognized as the primary source of principal ingredients, including flavonoids, magnesium, potassium, and iron. It also has antioxidant components, alpha-linolenic acid (omega 3), linoleic acid (omega 6), and oleic acid (omega 9) [21]. In vitro, the aqueous extract of pomegranate peels showed inhibition of COVID-19 virus replication [22]. It seems that consumption of pomegranate juice (PJ) can be applied for prophylactic and therapeutic approaches against COVID-19 [21]. At present, one RCT is aiming to evaluate the efficacy of PJ on inflammatory parameters, C-reactive protein (CRP), interleukin 6 (IL-6), erythrocyte sedimentation rate (ESR), and complete blood count (CBC) in mild to moderate COVID-19 patients (IRCT20150711023153N2) [23]. Further clinical studies must be performed to evaluate the effect of pomegranate in the current COVID-9 pandemic. In the present study, we investigated the clinical application of pomegranate in the treatment and prevention of different diseases.

## 2. Chemistry of *P. granatum* Fruit

### 2.1. Polyphenols

**Figure 1:**
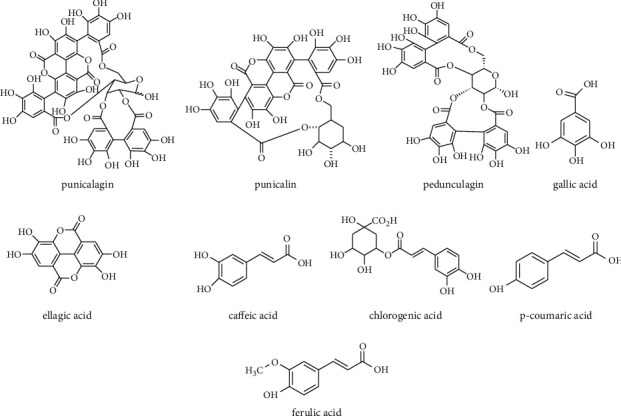
The main phenolic compounds in pomegranate.

The main classes of polyphenols identified in pomegranate are hydrolysable tannins including gallotannins, ellagitannins, gallagyl esters, hydroxycinnamic acids, and hydroxybenzoic acids. The major compound of ellagitannins is punicalagin (2,3-hexahydroxydiphenoyl-4,6 gallagylglucoside) which is mainly found in pericarp, bark, flowers, and seeds isolated by preparative HPLC [24]. In addition to punicalagin and its isomers, pomegranate contains punicalin A and B and pedunculagin isomers identified by MS and/or NMR. Gallic acid, ellagic acid, caffeic acid, chlorogenic acid, p-coumaric acid, aglycone, and ferulic acid have also been isolated from pomegranate by HPLC and NMR methods [25–27].  Figure 1 shows the main polyphenolic compounds from pomegranate.

### 2.2. Anthocyanosides

**Figure 2:**
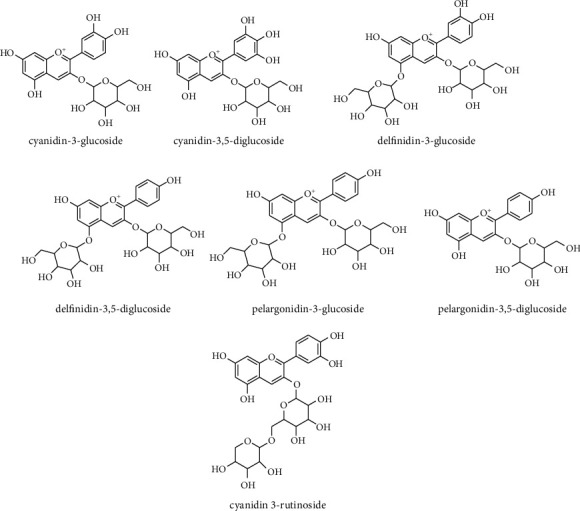
The main anthocyanoside compounds in pomegranate.

Anthocyanoside is another primary component present in the flower and fruit and is responsible for the red color of arils (Figure 2). Cyanidin-3-glucoside, cyanidin-3,5-diglucoside, cyanidin 3-rutinoside, cyanidin-pentoside, delfinidin-3,5-diglucoside, delfinidin-3-glucoside, pelargonidin-3-glucoside, and pelargonidin-3,5-diglucoside are the major anthocyanins detected in the arils, fruit, juice, and flowers elucidated by HPLC and NMR [8, 28].

### 2.3. Other Components

Pomegranate leaves and pericarp contain flavonols and flavones such as catechin, epicatechin, gallocatechin, kaempferol, quercetin, and apigenin, identified by IR and NMR [29]. Active compounds present in different parts of the pomegranate include alkaloids (e.g., pseudopelletierine, pelletierine, isopelletierine, methylpelletierine, 1-pelletierine, dl-pelletierine, and methylisopelletierines) identified by GLC-MS [30] and organic acids (e.g., citric acid, L-malic acid, oxalic acid, ascorbic acid, quinic acid, fumaric acid, tartaric acid, and succinic acid) elucidated by NMR and UHPLC-MS [31].

**Figure 3:**
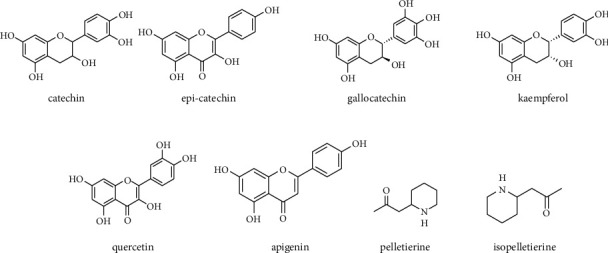
The main flavonoid and alkaloid compounds in pomegranate.

Lignans (e.g., furofuran, dibenzylbutyrolactone, and dibenzylbutane), minerals (e.g., Ca, P, K, N, Mg, and Na), ursane, oleanane triterpenes (e.g., triterpenic acids), and steroids are other active components isolated from pomegranate identified by LC-MS, HPLC-DAD, and GC-MS [32–35]. The main flavonoid and alkaloid compounds in pomegranate are shown in Figure 3.

## 3. Research Method

We conducted literature research in electronic databases (PubMed, Scopus, and ScienceDirect) to extract clinical studies on *Punica granatum* or pomegranate with no time limitation. The included search words were “*Punica granatum*,” “Pomegranate,” “Clinical trial,” “Clinical study,” “Blind,” or “Volunteer.”

## 4. Clinical Studies on Different Diseases

**Figure 4:**
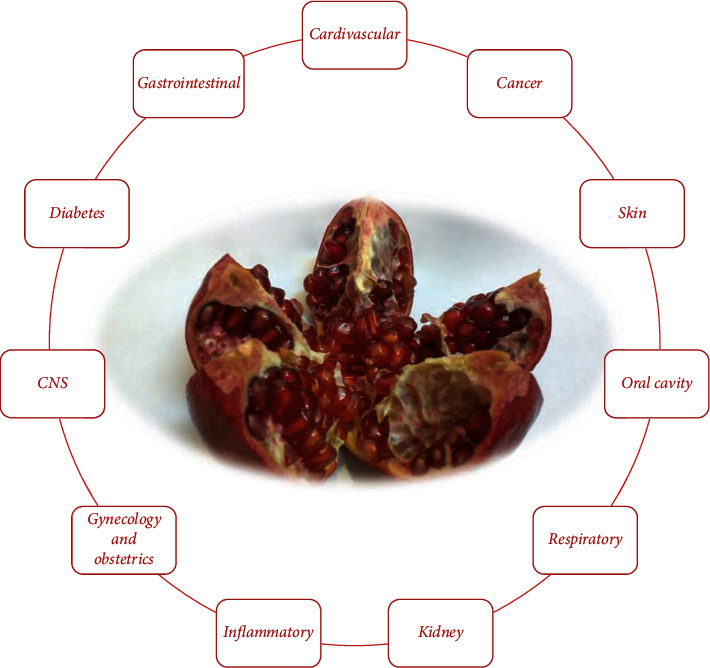
A summary of pomegranate clinical trials in different diseases.

Because of multiple pharmacological activities, pomegranate has been investigated by several clinical studies in a large variety of medical disorders. Different pomegranate activities include anti-inflammatory, antioxidant, and anticancer. Summary of clinical trials using *Punica granatum* (pomegranate) in different diseases is illustrated in Tables 1–4 and Figure 4.

### 4.1. Diabetes

#### 4.1.1. Fasting Blood Glucose

In animal studies, polyphenol and antioxidant rich fruits such as pomegranate have shown to stimulate beta cells to secrete insulin [82]. Several human studies have also evaluated the antidiabetic effects of pomegranate. The effect of pomegranate juice (PJ) in diabetic patients in reducing fasting blood glucose (FBG) may be associated with punicic acid, methanolic seed, and pomegranate peel extracts. The underlying mechanism responsible for this activity was reducing oxidative stress and lipid peroxidation and inhibiting or activating nuclear factor *κ*B and peroxisome proliferator-activated receptor *γ* [83]. In one quasi-experimental study on 55 diabetic patients, consumption of concentrated pomegranate juice (CPJ) (45 g/day for 3 month) showed no statistically significant effect on FBG [84]. In another randomized clinical trial (RCT), PJ consumption (250 ml/day for 12 weeks) by type 2 diabetic (T2D) patients did not affect FBG or homeostatic model assessment of insulin resistance (HOMA-IR) [85]. Moreover, consumption of CPJ (50 g/day of for 4 weeks) in T2D patients had no statistically significant effect on FBG [86]. Intake of pomegranate seed oil (PSO) (2000 mg/day for 8 weeks) had no effect on FBG and insulin resistance in 80 diabetic patients [87]. Similarly, wonderful variety pomegranate juice (WPJ) or pomegranate polyphenol extract (WPOMxl) consumption by 30 diabetic patients had no significant effects on FBG or HbA1c level [88]. Conversely, consumption of PSO (1 g/TID for 8 weeks) in 52 obese T2D patients significantly reduced FBG (*p*=0.008), but insulin, HbA1C, HOMA-IR, and HOMA-*β* did not show any significant changes [89]. Also, fresh PJ consumption (1.5 ml/kg) in 85 diabetic patients revealed significant reduction in FBG and insulin resistance and increased *β*-cell function (*p* < 0.05). This hypoglycemic effect was correlated with baseline FBG levels, as patients with lower FBG levels showed better hypoglycemic response (*p* < 0.05) [90]. In a randomized, double-blind, placebo-controlled trial on 40 patients with diabetes and myocardial infarction, administration of pomegranate extract of whole fruit (PEWF) (300 mg/BID for one month) significantly reduced FBG, postprandial glucose, and HbA1C levels (*p* < 0.05) [82]. In another study, consumption of PJ (200 ml/day for 6 weeks) by 50 T2D patients significantly reduced FBG (*p* < 0.001) [91]. Overall, the hypoglycemic effect of PJ or other compounds is limited due to the type of crop, harvest method, dosage, bioavailability, and duration of treatment.

#### 4.1.2. Oxidative Stress

One of the important factors in the pathogenesis of cardiovascular events in diabetic patients is hyperglycemia-induced oxidative stress. Several studies have shown the beneficial role of pomegranate on reducing oxidative stress and lipid peroxidation through direct neutralization of reactive oxygen species (ROS), upregulating antioxidant enzymes, and inhibition or activation of transcription factors such as nuclear factor *κ*B (NF-*κ*B) or peroxisome proliferator-activated receptor *γ* (PPAR*γ*). Various ingredients in pomegranate (e.g., punicalagin, ellagic, gallic, oleanolic, ursolic, and uallic acids) have antidiabetic effects. Also, antioxidant polyphenols including tannins and anthocyanins in the juice sugar fraction could be effective in T2D patients [83]. The antioxidant effects of polyphenol components found in green tea extract, pomegranate extract, and ascorbic acid were studied in uncomplicated T2D patients. Plasma malondialdehyde (MDA), a by-product of lipid peroxidation, was significantly decreased in the study group compared to placebo group (*p* < 0.001). Total glutathione (GSH) and antioxidant capacity (AOC) as markers of increased antioxidant potency were increased in the study group (*p* < 0.001) [92]. A pilot study investigated the antioxidant effects of pomegranate polyphenols in T2D patients. Consumption of pomegranate polyphenols (2 capsules/day of for 4 weeks) significantly reduced lipid peroxidation reactive metabolites (MDA and hydroxynonenal (HNE)) in the diabetic group compared to baseline (*p* < 0.05). However, there was no change in oxidized low-density lipoprotein cholesterol (LDL-C) level in either groups. These findings revealed that pomegranate polyphenols can decrease lipid peroxidation in diabetic patients with no effect on healthy volunteers [93]. In another study, consumption of CPJ (50 g/day for 4 weeks) in T2D patients increased the serum total antioxidant capacity (TAC) by 75% (from 381.88 ± 114.4 at baseline to 1501 ± 817 after administration) [86]. In an RCT, 12-week PJ consumption in T2D patients increased TAC (*p* < 0.05) and decreased MDA levels (*p* < 0.01), compared to the placebo group. But no statistically significant changes were observed in advanced glycated end-product (AGE) markers including carboxy methyl lysine (CML) and pentosidine [94]. In another RCT, consumption of PJ (200 ml/day for 6 weeks) in T2D patients decreased oxidized LDL and anti-oxidized LDL antibodies and increased serum TAC and arylesterase activity of paraoxonase [95]. In another study, PEWF (300 mg/BID for one month) significantly reduced total antioxidant activity, glutathione peroxidase, super-oxide dismutase, and glutathione reductase in patients with diabetes and myocardial infarction (*p* < 0.05) [82]. PJ consumption (50 ml/day for 3 months) significantly reduced lipid peroxides and thiobarbituric acid reactive substances (TBARS) levels, whereas sulfhydryl (SH) groups and paraoxonase 1 (PON1) activity were significantly increased. This study showed antioxidative activity of PJ on serum and on macrophages which could be beneficial for improvement of atherosclerosis in diabetic patients [96].

#### 4.1.3. Inflammation

CPJ consumption (50 g/day for 4 weeks) in T2D patients significantly decreased serum interleukin-6 (IL-6) (*p* < 0.05), but no changes were seen with tumor necrosis factor-*α* (TNF-*α*) and high-sensitivity C-reactive protein (hs-CRP) [86]. In another RCT, PJ consumption (250 ml/day for 12 weeks) in T2D patients reduced plasma hs-CRP and IL-6 levels by 32% and 30%, respectively (*p* < 0.05). After 12-week consumption of PJ, the mean plasma IL-6 and hs-CRP were significantly lower than those of the placebo group (*p* < 0.05) [85]. Also, plasma E-selectin concentration was significantly reduced in the PJ group compared to baseline and placebo group (*p* < 0.001 and *p* < 0.05, respectively). In the PJ group, NF-*κ*B p65 in peripheral blood mononuclear cell (PBMC) was significantly decreased (*p* < 0.01) and sirtuin 1 (SIRT1) was increased (*p* < 0.0001) compared to placebo group [97]. A pilot study investigated the anti-inflammatory effects of pomegranate polyphenols (2 capsules/day for 4 weeks) in T2D patients. Pomegranate polyphenols reduce lipid peroxidation and modulate liver enzymes in patients with T2DM with no effects in healthy controls [93]. Pomegranate has anti-inflammatory properties but studies with a longer duration and bigger sample size are required to further evaluate its effect.

#### 4.1.4. Lipid Profile

The effect of CPJ on lipid profile in T2D patients with hyperlipidemia (cholesterol ≥5.2 mmol/L or triacylglycerol ≥2.3 mmol/L) was studied. Consumption of CPJ (40 g/day for 8 weeks) remarkably reduced total cholesterol (TC), LDL-C, LDL-C/high-density lipoprotein cholesterol (HDL-C), and TC/HDL-C (*p* < 0.006,  *p* < 0.006,  *p* < 0.001,  and *p* < 0.001, respectively) with no significant change in triacylglycerol and HDL-C levels [98]. Also, consumption of PJ (200 ml/day for 6 weeks) by 50 T2D patients significantly reduced TC and LDL-C (*p* < 0.001), but there was no overall change in triglyceride (TG) and HDL-C [91]. The effects of polyphenol components (green tea extract, pomegranate extract, and ascorbic acid) on lipid profile were studied in uncomplicated T2D patients. In the study group, the LDL-C level showed statistically significant reduction, whereas the HDL-C level was increased compared to placebo group (*p* < 0.001 and *p* < 0.001, respectively). Moreover, total plasma GSH and AOC were found to be increased in the intervention compared with the control group [92]. Findings from an RCT revealed that consumption of PJ (200 ml/day for 6 weeks) in T2D patients significantly decreased TC and LDL-C levels compared to baseline. However, there was no difference in TC, TG, LDL-C, and HDL-C levels between the PJ and control groups [99]. In one quasi-experimental study on 55 diabetic patients, consumption of CPJ (45 g/day for 3 month) showed no statistically significant effects on TC and LDL-C [84]. Conversely, CPJ ingestion (50 g/day for 4 weeks) in T2D patients increased TC and HDL-C levels compared to baseline (*p* < 0.05) with no change in serum TG and LDL-C levels [86]. In a randomized double-blind clinical trial on 80 T2D patients, PSO (1000 mg/BID for 8 weeks) had no effect on TC, TG, HDL, and LDL-C [87]. In a recent double-blind, placebo-controlled randomized trial, administration of pomegranate peel extract (PoPEx) capsule (twice a day for 8 weeks) in 37 T2D patients significantly reduced plasma levels of TG and LDL-C/HDL-C, increased the HDL-C levels, and enhanced the plasma fatty acid composition [100]. Due to controversial findings obtained from clinical studies, a potential role of pomegranate on improving lipid profile in diabetic patients necessitates more evaluation.

#### 4.1.5. Blood Pressure

PJ is a rich source of soluble polyphenols including anthocyanins and tannins (e.g., ellagitannins (mostly punicalagin), ellagic acid, gallic acid, and catechins). These compounds have anti-inflammatory, antioxidant, anti-hyperlipidemic, and anti-hypertensive properties [99, 101]. It has been reported that consumption of CPJ (50 g/day for 4 weeks) in T2D patients had no effect on blood pressure [86]. On the contrary, consumption of PJ (200 ml/day for six weeks) significantly decreased systolic blood pressure (SBP) and diastolic blood pressure (DBP) (*p* < 0.001 and *p* < 0.05, respectively) in T2D patients. Also, SBP and DBP were lower in the PJ group compared to the control group (*p* < 0.02 and *p* < 0.03, respectively) [99]. Similarly, PoPEx administration for 8 weeks reduced both SBP and DBP in diabetic patients as was suggested in a recent RCT [100]. The anti-hypertensive effect of pomegranate has been indicated in scant studies, highlighting the need for more extensive results.

#### 4.1.6. Liver Enzymes

Intake of pomegranate polyphenols (2 capsules/day for 4 weeks) increased aspartate aminotransferase (AST) in healthy control group but decreased alanine aminotransferase (ALT) in diabetic patients (*p* < 0.05) [93]. This finding suggests that pomegranate can modulate liver enzymes in diabetic patients.

#### 4.1.7. Obesity

Central obesity is the most common cause of diabetes, cardiovascular disease, and hyperinsulinemia. Pomegranate is a flavonoid-rich fruit with antioxidant properties. The effects of PJ on insulin resistance, hs-CRP, and obesity were studied in an RCT on 50 T2D patients. After 8 weeks, a significant reduction in insulin resistance, body weight, and hip and waist circumference was observed in the study group (*p* < 0.05,  *p* < 0.01,  *p* < 0.05,  and *p* < 0.05, respectively), whereas serum glucose, HbA1C, and hs-CRP remained unchanged [102].

#### 4.1.8. Serum Cortisol and Thyroxine

Intake of fresh PJ (1.5 mL/kg) on 89 T2D patients showed remarkable lower levels of serum cortisol one hour after consumption (*p* < 0.0001) which was not related to FBG level or gender [103].

#### 4.1.9. Erythropoietin (EPO)

Administration of fresh PJ (1.5 ml/kg) reduced EPO in diabetic patients (but not in healthy subjects) three hours following consumption and was inversely correlated with FBG, but not with gender or age [104].

#### 4.1.10. Paraoxonase Enzyme Activity

PON1, an HDL-associated enzyme synthesized in the liver, is known to play an important role in prevention of LDL oxidation. Several studies have indicated reduced activity of PON1 in diabetic patients. Consumption of PJ (200 ml/day for 6 weeks) by 50 T2D patients significantly increased paraoxonase and arylesterase activity of PON1 (*p* < 0.001) which had positive and negative correlation with HDL-C and FBG levels, respectively [91].

Rock et al. explored the effects of WPJ (50 ml/day for 4 weeks) and WPOMxl (5 ml/day for 6 weeks) on PON1 and HDL association in 30 diabetic patients. They concluded that WPJ and WPOMxl delayed the development of atherosclerosis in diabetic patients through PON1 stabilization and increased the association of PON1 with HDL and its catalytic activity [88]. Likewise, PJ consumption (50 ml/day for 4 weeks) by 6 diabetic patients increased the capacity of HDL to bind recombinant paraoxonase-1 [105].

### 4.2. Cancer

Several in vitro and in vivo studies demonstrated the anticancer properties of pomegranate which inhibits the proliferation of tumor cells and induces their apoptosis. In this section, we review the clinical literature on colorectal, head and neck, and prostate cancers. Intake of pomegranate (2 capsules/day for 6-7 weeks) protected against mucositis- and dermatitis-induced radiotherapy in head and neck cancer [106]. In studies by Nuñez-Sánchez et al., ellagic acid and urolithin derivatives were found in colorectal cancer tissues following consumption of pomegranate extract (900 mg for 15 days) [107]. The same dosage of ellagitannin-containing pomegranate extract successfully modulated gene expression of TYMs, CD44, CDKN, and CTNNB in 35 patients with colorectal cancer [108]. Besides, the expression of microRNAs (a marker for colorectal cancer) on the same group of patients and same dosage of pomegranate extract revealed a statistically significant difference between control and treatment group [109]. Another study showed that pomegranate capsule (900 mg) reduced lipopolysaccharide-binding protein (LBP) levels, a marker of endotoxemia, in 57 patients with colorectal cancer [110]. Falsaperla et al. concluded that consumption of pomegranate (a capsule/day containing 180 mg of ellagic tannins) reduced neutropenia and toxicity induced by chemotherapy in hormone-refractory prostate cancer [111]. Urolithin, a metabolite of ellagitannins, was detected in prostate tissue following preoperative administration of PJ (200 ml/day for 3 days) in 63 patients with prostate cancer; however, no changes in the expression of MKi-67, c-Myc, or CDKN1A were observed [112]. In an RCT by Stenner-Liewen et al. on 102 patients with advanced prostate cancer, PJ consumption (500 ml/day and 250 ml/day for two sequential four weeks) revealed no significant difference between intervention and placebo groups in kinetics and pain scores of PSA serum levels [113].

The efficacy of pomegranate on PSA doubling time was investigated in an RCT on 183 patients undergoing primary therapy for prostate cancer. Patients were assigned to treatment (extract and juice) and placebo groups, and no statistically significant difference was observed in either groups [114]. Paller et al. concluded that consumption of 1 or 2 capsules/day, each one containing 1000 mg polyphenol equal to 8 oz of PJ for 18 months, reduced PSA doubling time in men with repeated prostate cancer [115]. Another study showed that pomegranate extract tablet (1000 mg containing 600 mg of polyphenol) for four weeks before radical prostatectomy could not lessen 8-hydroxy-20-deoxyguanosine (8-OHdG) levels, an oxidative stress biomarker, in 70 men [116]. Although, there are contradictions regarding pomegranate supplementation in cancer treatment, it could be a beneficial anticancer candidate due to components such as ellagic acid. Hence, more RCTs are still needed to determine the underlying molecular mechanism for pomegranate.

### 4.3. Cardiovascular Effects

#### 4.3.1. Hypertension

Juice, whole fruit, seed extract, seed oil, and flowers of *P. granatum* have been used in traditional Persian medicine as anti-hypertensive, cardioprotective (reduction in fibrosis), and anti-hyperlipidemic agents [117]. Anti-hypertensive properties of *P. granatum* are reported in several literatures. In a study by Aviram and Dornfield, PJ consumption (50 ml for 2 weeks) in 10 hypertensive patients reduced serum angiotensin-converting enzyme (ACE) levels (by 36%) and SBP (by 5%) which were attributed to antioxidative activity of pomegranate [118]. In a single-blind, placebo‐controlled clinical trial, intake of PJ (150 ml/day for 2 weeks) in 21 hypertensive subjects reduced SBP, DBP, and vascular endothelial adhesion molecule-1 (VCAM-1) (*p*=0.002,  0.038,  and 0.008, respectively), without any change on flow-mediated dilatation (FMD), intracellular adhesion molecule-1 (ICAM-1), and hs-CRP (*p* > 0.05) [119]. Asgary et al. evaluated the efficacy of PJ intake (150 ml/day following a 12-hour fasting) on 13 hypertensive patients. A significant reduction in SBP (*p*=0.013) and DBP (*p* < 0.010) was observed, but FMD, CRP, VCAM-1, ICAM-1, and IL-6 levels remained unchanged (*p*=0.172) [120]. Reduction of systolic, diastolic, and mean arterial blood pressure was reported in an RCT in which healthy subjects consumed 330 ml/day of PJ for four weeks [121]. Another RCT approved the previous study after drinking 500 ml/day of PJ for four weeks [122]. In short, most studies are inadequate, and we strongly suggest to use pomegranate with caution in hypertensive patients.

#### 4.3.2. Lipid-Lowering Effect

Statins or hydroxy-methyl-glutaryl-coenzyme A (HMG-CoA) reductase inhibitors are the first-line treatment in hypercholesterolemia. These drugs have numerous side effects including myalgia, myositis, rhabdomyolysis, myopathy, and diabetes mellitus [123]. In an RCT, a supplement containing 12 antioxidant fruits including pomegranate was given to 44 males (900 mg/TID for 4 weeks). A reduction in total plasma cholesterol and LDL levels (*p* < 0.05) and an increased level of HDL (*p* < 0.001) were detected in the supplement group following the intervention [124]. In another study, intake of PJ (200 ml/day) or lovastatin for 4 weeks on 60 patients with hypercholesterolemia generated the same results (*p* < 0.001) [125]. Contrary to previous studies, 400 mg pomegranate seed oil, twice daily for four weeks, does not affect TNF-*α* levels serum in dyslipidemic patients but could decrease TAG and TAG : HDL-C ratio [126, 127]. Hamoud et al. discovered that consumption of pomegranate extract pill (1 g/day) for two months caused attenuation in LDL-cholesterol levels and improvement in oxidative stress in hypercholesterolemic subjects [128]. Ultimately, as mentioned earlier, more clinical trials with larger sample size are necessary to ascertain the effect of pomegranate on hyperlipidemia.

#### 4.3.3. Metabolic Syndrome

Metabolic syndrome is a well-known cardiovascular risk factor recognized by dyslipidemia, hyperglycemia, hypertension, abdominal obesity, and proinflammatory states. In a study by Moazzen and Alizadeh, consumption of PJ (500 ml for one week) in 32 patients with metabolic syndrome decreased SBP, DBP, and blood hs-CRP but increased TG and very low-density lipoprotein cholesterol (VLDL) due to the high level of fructose [129]. In a clinical study on 23 females with metabolic syndrome who received 300 ml of PJ, the levels of arachidonic acid, lipid peroxidation, TBARS were decreased, whereas monounsaturated fatty acids (MUFA) and saturated fatty acids (SFA) were increased [130]. Many studies reported that consumption of grape (18 ml/day) and/or PJ (240 ml/day) by adolescents with metabolic syndrome for 1 month improved endothelial function and flow-mediated dilation and reduced inflammatory factors including sEselectin, sVCAM, sICAM-1, and IL-6 [131, 132]. Also, consumption of pomegranate concentrate (50 g for 8 weeks) in obese females with metabolic syndrome led to reduction of glucose, insulin, and insulin resistance and improved markers of metabolic syndrome and cardiac and respiratory endurance [133]. It is also reported that pomegranate extract reduced markers of platelet activation such as GPIIb-IIIa, p-selectin, and platelet-neutrophil aggregation in 4 subjects with metabolic syndrome [134]. Despite few numbers of patients and short period of treatment, we concluded that consumption of PJ possesses beneficial effects on metabolic syndrome.

#### 4.3.4. Coronary Heart Disease

Ischemic heart disease and oxidative stress following ischemia reperfusion injury cause lipid peroxidation in lipoproteins and arterial macrophages [135]. In a randomized double-blind clinical trial on 15 patients with carotid artery stenosis, consumption of PJ (50 ml containing 1.5 mmol of total polyphenols for a year) reduced SBP and carotid intima-media thickness and inhibited lipid peroxidation in LDL and serum which was attributed to the antioxidant activity of polyphenols [136]. Also, in another randomized clinical trial on 289 patients with coronary heart disease, treatment with PJ (240 ml/day for 18 months) lowered the progression of carotid intima-media thickness in patients with increased levels of oxidative stress [137]. Comparably, the effect of PJ intake (240 ml/day for 3 months) in 45 patients with coronary heart disease and myocardial ischemia was reported in a randomized, double-blind, placebo-controlled clinical trial. The results showed an improvement in myocardial ischemia and myocardial perfusion without any effects on blood sugar, blood pressure, weight, and hemoglobin A1c [138]. In a study by Razani et al., a reduction in intensity and duration of angina pectoris and improvement in myocardial ischemia and reperfusion injury in patients with ischemic heart disease were observed following PJ administration [139]. Based on the above data, the antioxidant features of pomegranate juice extract contribute to its robust protective effects against coronary heart disease.

#### 4.3.5. Obesity

Obesity is a common health concern facing our society with major adverse comorbidities including cardiovascular disease, dyslipidemia, hypertension, T2D, respiratory disorders, and cancer. In a clinical study on 38 obese female with dyslipidemia, consumption of 500 mg pomegranate peel extract reduced the levels of serum TC, LDL-C, TG, and hs-CRP and improved serum lipid profile, SBP, and HDL-C [140]. In a randomized clinical trial on 49 overweight-obese subjects with hyperlipidemia, pomegranate extract (450 mg) was administrated in two individual doses (D1 (1 capsule/day) or D2 (4 capsules/day) for 3 weeks). The consumption of pomegranate extract at a dose of D2 (1.8 g/day) modulated the gut microbiota, reduced plasma LBP, and thus decreased metabolic endotoxemia and cardiovascular risk [141]. In two pilot clinical studies, the antioxidant activity and safety of pomegranate ellagitannin-enriched polyphenol extract were assessed in overweight individuals with increased waist size. There was a significant reduction in TBARS related to cardiovascular disease risk, and no serious adverse effects were observed [142]. Furthermore, a double-blind cross-over design on 14 overweight individuals showed a significant reduction in thiol (SH) levels 0.5 (*p* < 0.05), 1 (*p* < 0.05), and 2 hours (*p* < 0.01) after consumption of high-fat meals plus antioxidant beverage 1 (HFM-AB1; apple, grape, blueberry, and pomegranate juices and grape skin, grape seed, and green tea extracts). Plasma total radical-trapping antioxidant parameter (TRAP) (2 h, *p* < 0.001) and urinary ferric reducing antioxidant potency (0–8 h, *p* < 0.01) were remarkably increased [143]. In another study, 48 obese and overweight patients received pomegranate extract (1000 mg) or a placebo. After 30 days, the levels of glucose, insulin, LDL-C, TC, IL-6, MDA, and hs-CRP were decreased [144]. Grape/pomegranate extract dietary supplement had no mitigation effects on 20 adults with abdominal obesity. But it improved insulin sensitivity when consumed 10 hours prior to the administration of oral glucose tolerance test (OGTT) [145]. Also, in a randomized, double-blind clinical trial on 20 obese subjects, treatment with PJ (120 ml) reduced adiposity but did not modify insulin secretion and sensitivity [146]. Park et al. indicated that the intake of pomegranate vinegar (700 *μ*g ellagic acid/200 mL/day and 1.5 g acetic acid for 8 weeks) by 78 overweight women led to a reduction in visceral adipose tissue and an increase in AMP-activated protein kinase phosphorylation [147]. In general, consumption of pomegranate juice/extract may prevent obesity and its related disorders.

### 4.4. Oral Cavity

#### 4.4.1. Dental Plaque and Gingivitis

Pomegranate extract acts as an anti-inflammatory compound and free radical scavenger capable of reducing macrophage oxidative stress and lipid peroxidation. It protects against gingivitis through direct antioxidant activity of flavonoid content and indirect effects via increasing free radical scavengers [148]. Several studies have evaluated the effect of pomegranate on oral cavity disorders. In a randomized double-blind controlled trial on 84 patients, pomegranate peel extract lozenge showed significant reduction in gag reflex within dental procedures [149]. The effect of *Achyranthes aspera*, 0.2% aqueous chlorhexidine gluconate, and *P. granatum* mouthwash (140 ml as a daily rinse after breakfast and before sleeping) on salivary *Streptococcus mutans* level was assessed in 60 children. After seven days, all of the respective mouthwashes revealed statistically significant decline in *S. mutans* and plaque index levels (*p* < 0.01 and *p* < 0.05, *p* < 0.001 and *p* < 0.05, and *p* < 0.01 and *p* < 0.05, respectively). Chlorhexidine mouthwash had marginally better results in reducing *S. mutans* levels, and *P. granatum* mouthwash showed more antimicrobial effects than *A. aspera* [150]. Efficacy of *P. granatum*, *Terminalia chebula*, and *Vitis vinifera* mouthwashes (10 ml once daily after dinner for 15 days) on salivary *S. mutans* levels was investigated in a randomized clinical double-blinded study on 80 children subclassified into 4 groups. *P. granatum* revealed maximum reduction in *S. mutans*, but there was no statistically significant difference between all groups [151]. Another single-blind randomized controlled trial carried out in 20 healthy individuals also revealed statistically significant reduction in mean plaque of *S. mutans* in both chlorhexidine and *P. granatum* groups at baseline and on the 7th day follow-up. But there was no statistically significant difference in mean plaque *S. mutans* between two groups at the 7th day of follow-up [152]. In another randomized, controlled, double-blind clinical trial on 35 students, *P. granatum* Linn. mouthwash (6.25%) (twice a day for 14 days) showed a significant reduction in the oral streptococci count [153]. Also, the results of one study on 60 healthy patients indicated that hydroalcoholic extract of *P. granatum* is more effective than both chlorhexidine and distilled water against dental plaque microorganisms (reduction in the colony forming units per milliliter (CFU/ml) by 84%, 79%, and 11%, respectively) [154]. These studies showed that *P. granatum* can be used as an alternative to chlorhexidine mouthwash against plaque *S. mutans*. A gel containing 10% *P. granatum* extract was evaluated in a cross-over, double-blind study on 23 volunteers for 2 phases of 21 days each. The results showed no statistically significant difference between placebo and test gel for visible plaque index (VPI) and gingival bleeding index (GBI). This study did not provide antiplaque and antigingivitis effects for *P. granatum* gel [155]. Another randomized, controlled, double-blind clinical trial conducted on 35 students showed lack of efficacy of *P. granatum* mouthwash (6.25%) (twice a day for 14 days) for the management of dental biofilm and gingivitis [153]. On the contrary, several studies are available on positive effects of pomegranate mouthwash or gel in reduction of dental plaque and gingival inflammation. In a double-blind randomized controlled trial, the efficacy of pomegranate and chamomile mouth rinse was assessed in 55 patients who suffered from chronic gingivitis for 15 days. The patients were randomly divided into three groups: (1) control group: chlorhexidine 0.12% solution mouthwash (*n* = 18); (2) chamomile extract mouthwash (*n* = 19); and (3) pomegranate extract mouthwash (*n* = 18). The chamomile and pomegranate extracts reduced gingival bleeding due to their anti-inflammatory and antimicrobial activities similar to chlorhexidine 0.12% [156]. Another double-blind, single-center, controlled clinical trial on 40 adolescents suggested that pomegranate extract mouthwash (twice daily for 90 days) reduced the mean plaque and gingival index values, as well as total salivary proteins without any adverse effects [157]. A randomized, single-blinded controlled trial was conducted on 32 young adults to examine the effects of pomegranate extract mouthwash (thrice daily for 4 weeks) on gingivitis. It reduced total protein, aspartate aminotransferase activities, alpha-glucosidase activity and increased the antioxidant enzyme ceruloplasmin activities and radical scavenging potency. These data indicated the potential use of pomegranate extracts in toothpastes and mouthwashes [158]. Furthermore, a double-blind clinical trial in 104 patients with mild to moderate gingivitis showed that the effect of pomegranate mouthwash (twice daily for 1 month) was comparable to two commonly used herbal mouthwashes (Persica and Matrica) [159]. In a clinical study, 20 patients with chronic gingivitis used pomegranate or chlorhexidine mouthwash (10 ml twice daily for 15 days). A significant improvement in bleeding and gingivitis score was seen in the pomegranate group with no reduction in plaque scores [160]. Also, the effect of *P. granatum* var. *pleniflora* mouthwash (10 ml every night for 2 weeks) was evaluated in 80 patients with diabetes mellitus and gingivitis. After 2 weeks, modified gingival index in Golnaar mouthwash was more effective than chlorhexidine (*p*=0.039) [161]. In a controlled, single-blind, randomized study, the effect of herbal extracts including *P. granatum* (pomegranate), *Piper nigrum* (black pepper), and detoxified copper sulfate versus chlorhexidine was evaluated in 30 patients with chronic periodontitis. Herbal extracts revealed significant reduction in plaque index (PI) but had no effect on sulcus bleeding index (SBI) [162]. Also, the antiplaque effect of pomegranate mouthwash (twice daily for 5 days) was evaluated in 30 periodontally healthy volunteers. The PI significantly increased in all groups (pomegranate, chlorhexidine, and distilled water) (*p* < 0.01) with no statistically significant difference between the pomegranate and chlorhexidine groups. In vitro assay revealed efficacy of pomegranate extract against *Aggregatibacter actinomycetemcomitans*, *Porphyromonas gingivalis*, and *Prevotella intermedia* [163]. Another randomized clinical study in 40 patients with chronic gingivitis also showed that a pomegranate extract-containing gel along with mechanical debridement for 21 days could remarkably ameliorate all the clinical and microbiological indices [164]. In another RCT on 80 healthy subjects, *P. granatum* extract gel decreased the inflammatory markers including IL-1*β* and IL-8 serving as an adjunct to mechanical therapy for the treatment of gingivitis [165].

#### 4.4.2. Stomatitis

In several studies, *P. granatum* gel has been proven to be effective in the management of aphthous stomatitis. In a double-blind clinical trial, 60 patients with mild aphthous stomatitis randomly received *P. granatum* muco-adhesive gel, Triadent oral paste, and placebo (three times a day). The pain elimination time in *P. granatum* group was lower than that in placebo group (*p*=0.002); however, Triadent and placebo groups had no difference (*p*=0.08). The duration of wound healing in *P. granatum* group was lower than that in placebo and Triadent groups (*p*=0.001 and *p*=0.04, respectively) [166]. In another randomized, double-blind, placebo-controlled study on 40 patients with recurrent aphthous stomatitis (RAS), topical *P. granatum* gel 10% significantly reduced pain elimination time (*p* < 0.001), duration of complete healing (*p* < 0.001), and visual analog scale score (*p* < 0.001), compared to placebo [167]. Furthermore, in a randomized, double-blind study on 56 patients with RAS, the pomegranate peel extract gel (twice daily for one week) showed more efficacy in reducing pain (*p* < 0.001), ulcer size (*p* < 0.001), and duration of ulcer healing (*p* < 0.001) [168]. In a double-blind study on 210 participants with minor RAS, the alcoholic and water extracts of *P. granatum* var. *pleniflora* reduced the total time of complete treatment [169]. The antifungal effects of *P. granatum* Linne gel (three times a day for 15 days) were explored against candidiasis-related denture stomatitis in a double-blind study on 60 patients. Yeasts were not detected in 25 (83.3%) patients of miconazole group and 23 (76.7%) patients of pomegranate group [170].

#### 4.4.3. Periodontitis

In a preliminary study, scaling and root planning with subsequent subgingival delivery of *Centella asiatica* and *Punica granatum* in the form of biodegradable chips reduced clinical signs of chronic periodontitis in 20 patients [171]. In another study on 15 patients with periodontitis, adjunctive local treatment with *C. asiatica* and *P. granatum* extracts suggested a significant improvement in clinical parameters of chronic periodontitis and IL-1 levels compared to standard supportive periodontal therapy [172].

## 5. Conclusion

Several phytochemical constituents including polyphenolics, flavonoids, anthocyanosides, alkaloids, lignans, and triterpenes have been reported from the plant. The major pharmacological effects of pomegranate reported in randomized clinical trials pertain to diabetes, cardiovascular disease, oral cavity disorders, endocrine disorders, and cancer. The present review has provided an insight into the traditional applications of the plants, and some of them have been validated by scientific evidence, particularly their applications as treatment of cardiovascular and endocrine diseases. *Punica granatum* and its components have multiple pharmacological effects and clinical applications. Large clinical trials have looked into its therapeutic activities against inflammation, cardiovascular diseases (e.g., hyperlipidemia and hypertension), endocrinopathies (e.g., diabetes), and cancer. However, the underlying molecular mechanism of pomegranate is yet to be determined by more RCTs.

In diabetes, the hypoglycemic effect of the pomegranate juice or other compounds is limited, but its antioxidative effect has been largely discussed in this disorder. As suggested by one study, pomegranate also exerts a modulating effect on liver enzymes in diabetic patients.

Moreover, consumption of pomegranate juice has shown to have preventive roles on obesity, metabolic syndrome, and coronary heart disease (which is believed to be due to its potent antioxidant properties). The beneficial effects of pomegranate mouthwash or gel on periodontitis, gingivitis, and stomatitis are likely related to its anti-inflammatory, antioxidant, and antimicrobial activities. Other benefits of pomegranate suggested by small clinical studies include gastrointestinal system (e.g., anorexia and inflammatory bowel disease (IBD)), central nervous system (e.g., anxiety during dental procedures in children, ischemic stroke, and memory dysfunction in heart surgery), reproductive system (e.g., heavy menstrual bleeding of endometrial origin, uterine leiomyoma-related menorrhagia, polycystic ovarian syndrome (PCOS), and nausea and vomiting during pregnancy), rheumatic disorders (e.g., reactive arthritis (RA) and knee osteoarthritis), dermatologic disorders (e.g., striae distensae, facial photodamage, ultraviolet-induced pigmentation, and skin erythema), and renal diseases, among others. Consequently, further well-designed clinical trials are warranted to establish thorough clinical applications and therapeutic role of pomegranate especially in COVID-19.

## Figures and Tables

**Table 1:** Summary of clinical trials using *Punica granatum* (pomegranate) in endocrine disorders.

Disease	Treatment	No. of patients	Study design	Dosage/duration	Outcomes	References
Erectile dysfunction	(i) PJ (ii) Placebo juice	61	Double‐blind, RCT	8 ounces daily/two 4-week treatment periods separated by a 2-week washout	Improvement of GAQ scores.	[36]
Idiopathic central precocious puberty	(i) Pomegranate extract juice + GnRH analog (ii) Placebo + GnRH analog	225	Double‐blind, RCT	100 ml daily/3 months	Improvement of bone age, growth velocity and height standard deviation score.	[37]
Males with poor semen quality	(i) Tablets containing extract of pomegranate fruit and freeze-dried rhizome of greater galangal (iii) Placebo tablets	66	Double‐blind, RCT	4 tablets with extract of *P. granatum* and 4 tablets with *A. galanga* daily/3 months	Increase in the total number of motile spermatozoa in plant extracts versus placebo (*p*=0.026).	[38]
Non-alcoholic fatty liver disease	(i) PJ (ii) Orange juice	65	Un-blinded, RCT	250 ml daily/12 weeks	Increase in TAC in the pomegranate group (*p* < 0.01).	[39]

*Gynecology and obstetrics*						
Obese premenopausal women with non-alcoholic fatty liver disease	(i) Xanthigen™ (brown marine algae fucoxanthin + PSO) (ii) Placebo	151	Double‐blind, RCT	TDS/16 weeks	Reduction of weight, body, and liver fat content, and improvement of LFT in non-diabetic obese women.	[40]
Heavy menstrual bleeding of endometrial origin	(i) PG (ii) TA	76	Double‐blind, RCT	500 mg PG every 6 h or 500 mg of TA/5 consecutive days from the first day of menses for 3 cycles	Reduction in the duration of bleeding and improvement of QoL and hematological assessments.	[41]
Uterine leiomyoma-related menorrhagia	PG	19	Pilot study (before/after style)	5 ml syrup TDS for 7 days starting from the onset of heavy bleeding/3 consecutive menstrual periods	Reduction of uterine fibroid size and leiomyoma bleeding and improvement of the QoL.	[42]
Polycystic ovarian syndrome	(i) SPJ, PJ, and SB (ii) PB	92	Triple-blinded, RCT	2 L weekly/8 weeks	Improvement of insulin resistance, insulin, testosterone level, BMI, weight, and waist circumference in PCOS.	[43]
Polycystic ovarian syndrome	(i) SPJ, PJ, and SB (ii) PB	92	Triple-blinded, RCT	300 ml daily/8 weeks	Improvement of metabolic, oxidative, inflammatory, and BP consequences in PCOS.	[44]
Vasomotor symptoms of menopause	(1) Black cohosh (2) Multibotanical (black cohosh, alfalfa, chaste tree, dong quai, false unicorn, licorice, oats, pomegranate, Siberian ginseng, boron) (3) Multibotanical plus dietary soy counseling (4) Conjugated equine estrogen with or without medroxyprogesterone acetate (5) Placebo	351	Double‐blind, RCT	Four capsules daily/one year	Black cohosh alone or in combination with multibotanical product had little effect on alleviation of vasomotor symptoms.	[45]
Menopausal symptoms	(i) Pomegranate seed oil (ii) Placebo	81	Double‐blind, RCT	Two capsules daily/12 weeks	PGS in postmenopausal women could not show reduction in hot flashes during a 12-week period.	[46]

*Rheumatic disorders*						
Rheumatoid arthritis	POMx	6	Pilot study (open-labeled)	10 ml daily/12 weeks	Reduction of DAS28 in RA patients due to its antioxidant effects.	[47]
	(i) POMx (ii) Placebo	55	Double‐blind, RCT	2 capsules of 250 mg POMx or cellulose/8 weeks	Improvement of disease activity and some blood biomarkers of inflammation and oxidative stress in RA patients.	[48]
Knee osteoarthritis	(i) PJ (ii) Control	38	Parallel-RCT	200 ml daily/6 weeks	Improvement of physical function and stiffness and antioxidant properties in patients with knee OA.	[49]
	(i) Pomegranate peel, hydro alcoholic extract along with standard treatment (ii) Placebo along with standard treatment	60	Double‐blind, RCT	500 mg twice daily/8 weeks	Reduction of pain and improvement of symptoms in women with knee OA.	[50]

*Renal disorders*						
Nephrolithiasis	Pomegranate polyphenol extract	30	Pilot study	1000 mg daily/90 days	An increase in PON1 activity in RSFs with a trend toward reducing in SSCaOx.	[51]
Hemodialysis	(i) PJ (ii) Placebo juice	101	Double‐blind, RCT	100 ml three times a week/one year	Improvement of clinical outcomes, cardiovascular risk factors, innate immunity and reduced incidence of hospitalization due to infections.	[52]
	(i) PJ (ii) Placebo juice	27	Double‐blind, RCT	100 ml single-dose during the first hour of a dialysis session	Improvement of oxidative stress and inflammation induced by IV iron during dialysis.	[53]
	(i) PJ (ii) Placebo juice	101	Double‐blind, RCT	100 ml three times a week/one year	Reduction of systolic blood pressure and improved lipid profile.	[54]
	(i) PJ (ii) Pomegranate extract	24	Open-label, cross-over clinical trial	100 ml of juice before each dialysis or 1,050 mg of extract daily/12 weeks	No effect on inflammation or oxidative stress markers, lipid profile and blood pressure.	[55]
	(i) POMx (ii) Placebo	33	Double‐blind, RCT	1000 mg capsule 7 days/week/6 months	Reduction of blood pressure and improvement of antioxidant activity, but had no effect on other markers of cardiovascular risk, physical function, or muscle strength.	[56]
	(i) PJ (ii) Control: usual care	41	Randomized cross-over trial	100 ml after dialysis session three times a week/8 weeks	Improvement of lipid profile, blood pressure, oxidative stress, and inflammation.	[57]

**Table 2:** Summary of clinical trials using *Punica granatum* (pomegranate) in gastrointestinal disorders.

Disease	Treatment	No. of patients	Study design	Dosage/duration	Outcomes	References
Non-pathogenic anorexia	(i) Appetizer syrup: *Kismis* (*Vitis vinifera* L.), *Pipalli* (*Piper longum* L.), *Anar* (*Punica granatum* L.), *Amla* (*Emblica officinalis* Gaertn), etc. (ii) Placebo syrup	100	Double‐blind, RCT	5 ml twice daily/2 months	Improved abdominal fullness, general desire to eat, and satiety in the intervention group compared to placebo syrup.	[58]

Nausea and vomiting during pregnancy	(i) Pomegranate and spearmint syrup plus vitamin B6 (ii) B6 tablets	55	Un-blinded, RCT	Syrup: 5 cc TDS Control: 20 mg TDS/1 week	Reduction of PUQE-24 scores in the syrup group compared to control group (*p*=0.001).	[59]

Diarrhoea-predominant irritable bowel syndrome	(i) Ayurvedic herbal compound: *Murraya koenigii* (curry), *Punica granatum* (pomegranate), and *Curcuma longa* (turmeric) (ii) Placebo	22	Double‐blind, cross-over RCT	Twice daily/4 weeks followed by a one week wash out period	No significant improvement was observed in IBS-D symptoms compared to placebo.	[60]
	(i) Traditional Chinese medicine containing 11 herbs including *Punica granatum* (ii) Placebo	119	Double‐blind, RCT	Twice daily/8 weeks	No significant difference was observed in symptom and QoL scores between two groups.	[61]

Inflammatory bowel disease	(i) PJ (ii) Placebo	36	Double‐blind, RCT	125 ml twice daily/12 weeks	Reduction of fecal calprotectin levels in IBD patients.	[62]

Ulcerative colitis	(i) *Punica granatum* peels aqueous extract syrup (ii) Placebo syrup	62	Double‐blind, RCT	6 g of dry peel daily/4 weeks	Improvement of symptoms in UC patients.	[63]
	(i) *Punica granatum* peels aqueous extract (ii) Placebo	62	Double‐blind, RCT	6 g daily/4 weeks	Patients with hot temperament showed higher response to treatment in comparison to cold temperament.	[64]

**Table 3:** Summary of clinical trials using *Punica granatum* (pomegranate) in dermatologic disorders.

Striae distensae	Oil-in-water cream containing *Punica granatum* seed oil and *Croton lechleri* resin extract	20	Non-randomized study	Once daily on the hip area/6 weeks	Improvement of striae , dermis thickness, hydration and elasticity values.	[65]

Effects on skin biophysical parameters	(i) Fixed *Polypodium leucotomos*/pomegranate combination (PPmix®) (ii) *Polypodium leucotomos* alone (Fernblock®)	40	Double‐blind, RCT	480 mg daily/3 months	Fixed *Polypodium leucotomos*/pomegranate combination improved skin biophysical parameters more than *Polypodium leucotomos* alone in adult Caucasians.	[66]

Facial photodamage	(i) Vitaphenol skin cream containing green and white teas, mangosteen, and pomegranate extract (ii) Placebo cream	20	RCT	Twice daily/60 days	Combination of three antioxidants had an additive efficacy in improvement of facial photodamage.	[67]

Ultraviolet-induced pigmentation	(i) High-dose POMx (ii) Low-dose POMx (iii) Placebo	37	Double‐blind, RCT	(i) 200 mg daily ellagic acid (2 tablets daily)/4 weeks (ii) 100 mg daily ellagic acid (2 tablets daily)/4 weeks (iii) 0 mg daily ellagic acid (2 tablets daily)/4 weeks	Orally ellagic acid-rich pomegranate extract had a protective effect against slight sunburn due to UV irradiation.	[68]

Skin erythema and melanin	(i) Topical microemulsion (O/W) of POMx (ii) Placebo microemulsion (without extract)	11	Single-blinded, clinical study	At night on cheeks/12 weeks	Active microemulsion revealed a significant effect on skin erythema and melanin (*p* < 0.05).	[69]

UVB-induced erythema and changes the skin microbiome	(i) POMx (ii) PJ (iii) Placebo	74	Open-label RCT	(i) 1000 mg daily/12 weeks (ii) 8 oz daily/12 weeks (iii) 8 oz daily/12 weeks	Daily oral pomegranate intake can be helpful to protect against UVB-induced skin damage.	[70]

Diabetic foot ulceration	(i) Control (ii) *Punica granatum* extract (iii) *Propolis* extract	60	Pretest/posttest control quasi- experimental design	—	*Punica granatum* and *Propolis* are an effective extract to control the diabetic foot ulceration.	[71]

Non-healing chronic ulcer	2% (w/w) PGHF	1	Case study	Once a day/10 weeks	PGHF (an alternative agent in wound healing treatment).	[72]

Herpes zoster pain	Hot water extracts of a herbal formula containing *Ganoderma lucidum* and WTMCGEPP (*Wisteria floribunda* 0.38, *Trapa natans* 0.38, *Myristica agrans* 0.38, *Coix lachryma-jobi* 0.75, cultivated *Ganoderma lucidum* 0.75, *Elfuinga applanata* 0.38, tissue cultured *Panax ginseng* 0.3, and *Punica granatum* 0.38: numerals designate dry weight gram/dose)	5	Pilot clinical trial	Several doses daily	Increase in treatment response.	[73]

Dandruff	Antidandruff shampoo (Zinc-PCA and piroctone olamine in combination with *Punica granatum* L., *Rosmarinus officinalis* L., *Matricaria chamomilla* L., *Urtica dioica* L., *Mentha piperita* L., and *Salvia officinalis* L. methanolic extracts)	30	Clinical trial	3 times a week/2 months	Reduction of dandruff with low side effects.	[74]

**Table 4:** Summary of clinical trials using *Punica granatum* (pomegranate) in other disorders.

Disease	Treatment	No. of patients	Study design	Dosage/duration	Outcomes	References
Chronic obstructive pulmonary disease (COPD)	(i) PJ (ii) Synthetic orange-flavoured drink	30	Double‐blind, RCT	400 ml daily/5 weeks	No useful effect in patients with COPD.	[75]

Anxiety during dental treatment in children	Group I: Pepsi Cola + 0.75 mg/kg midazolam Group II: 10% sodium citrate + 0.75 mg/kg midazolam Group III: PJ + 0.75 mg/kg midazolam Group IV: grape fruit juice + 0.75 mg/kg midazolam Group V: 0.75 mg/kg midazolam orally	75	Double‐blind, RCT	Two milliliter each of compounds added to midazolam in equal volumes of 15 mg/3 ml	Increase in the effectiveness of sedation with addition of sodium citrate to the midazolam.	[76]

Ischemic stroke	(i) Pomegranate polyphenol pills (ii) Placebo pills	16	Double‐blind, RCT	Twice per day/one week	Improvement of cognitive and functional recovery after stroke.	[77]

Infants with intrauterine growth restriction	(i) PJ (ii) Placebo juice	55	Double‐blind, RCT	8 oz daily/until delivery	PJ altered white matter organization and functional connectivity in the infant brain.	[78]

Memory dysfunction in heart surgery	(i) POMx pills (ii) Placebo pills	10	Pilot study	2 pills daily/7 weeks	Improvement of memory retention skill after surgery.	[79]

Illnesses related to stress	OCTA© compound mixture of eight herbs including *Punica granatum*	17	Open-label and uncontrolled clinical trial	30 ml daily/3 months	Improvement of perceptions of stress and quality of life.	[80]

Hypercholesterolemia	(i) Malas variety of PJ (ii) Black variety of PJ (iii) Lovastatin	36	Clinical study	200 ml daily/4 weeks	Reduction of plasma TC and LDL (*p* < 0.01).	[81]

## Data Availability

No raw data were used to support this study.

## Abbreviations

FBG: Fasting blood glucose
HOMA-IR: Homeostatic model assessment of insulin resistance
RCT: Randomized clinical trial
T2D: Type 2 diabetes
CPJ: Concentrated pomegranate juice
WPJ: Wonderful variety pomegranate juice
WPOMxl: Pomegranate polyphenol extract
PEWF: Pomegranate extract of whole fruit
ROS: Reactive oxygen species
NF- *κ*B: Nuclear factor *κ*B
PPAR*γ*: Peroxisome proliferator-activated receptor *γ*
MDA: Malondialdehyde
GSH: Total glutathione
AOC: Antioxidant capacity
HNE: Hydroxynonenal
LDL-C: Low-density lipoprotein cholesterol
TAC: Total antioxidant capacity
AGE: Advanced glycated end-product
CML: Carboxy methyl lysine
TBARS: Thiobarbituric acid reactive substances
SH: Sulfhydryl
PON1: Paraoxonase 1
IL-6: Interleukin-6
TNF-*α*: Tumor necrosis factor-*α*
hs-CRP: High-sensitivity C-reactive protein
PBMC: Peripheral blood mononuclear cell
SIRT1: Sirtuin 1
TC: Total cholesterol
HDL-C: High-density lipoprotein cholesterol
TG: Triglyceride
PoPEx: Pomegranate peel extract
SBP: Systolic blood pressure
DBP: Diastolic blood pressure
AST: Aspartate aminotransferase
ALT: Alanine aminotransferase
EPO: Erythropoietin
ACE: Angiotensin-converting enzyme
FMD: Flow-mediated dilatation
VCAM-1: Vascular endothelial adhesion molecule-1
ICAM-1: Intracellular adhesion molecule-1
HMG-CoA: Hydroxy-methyl-glutaryl-coenzyme A
VLDL: Very low-density lipoprotein cholesterol
MUFA: Monounsaturated fatty acids
SFA: Saturated fatty acids
HFM-AB1: High-fat meals plus antioxidant beverage 1
TRAP: Total radical-trapping antioxidant parameter
OGTT: Oral glucose tolerance test
PI: Plaque index
SBI: Sulcus bleeding index
RAS: Recurrent aphthous stomatitis
WAIS-R: Wechsler Adult Intelligence Scale-Revised
MoCA: Montreal cognitive assessment
IIEF: International index of erectile function
GAQ: Global assessment questionnaires
BDNF: Brain-derived neurotrophic factor
Zinc-PCA: Zinc L-pyrrolidone carboxylic acid
GnRH: Gonadotropin-releasing hormone
IGF-1: Insulin-like growth factor 1
PHN: Postherpetic neuralgia
PGHF: *P. granatum* peel ethanolic extract hydrogel-based formulation
PSO: Pomegranate seed oil
LFT: Liver function test
PG: Persian Golnar
TA: Tranexamic acid
QoL: Quality of life
RSFs: Recurrent stone formers
SSCaOx: Supersaturation of calcium oxalate
SPJ: Synbiotic pomegranate juice
SB: Synbiotic beverage
PJ: Pomegranate juice
PB: Placebo beverage
BMI: Body mass index
PCOS: Polycystic ovarian syndrome
BP: Blood pressure
TDS: Three times a day
PUQE-24: 24-hour pregnancy-unique quantification of emesis
IBS-D: Diarrhoea-predominant irritable bowel syndrome
POMx: Pomegranate extract
RA: Rheumatoid arthritis
DAS28: Disease Activity Score 28
OA: Osteoarthritis
IBD: Inflammatory bowel disease
UC: Ulcerative colitis
PGS: Pomegranate seed oil
IV: Intravenous.
